# Role of Glycosyltransferases Modifying Type B Flagellin of Emerging Hypervirulent *Clostridium difficile* Lineages and Their Impact on Motility and Biofilm Formation*[Fn FN2]

**DOI:** 10.1074/jbc.M116.749523

**Published:** 2016-10-04

**Authors:** Esmeralda Valiente, Laura Bouché, Paul Hitchen, Alexandra Faulds-Pain, Mario Songane, Lisa F. Dawson, Elizabeth Donahue, Richard A. Stabler, Maria Panico, Howard R. Morris, Mona Bajaj-Elliott, Susan M. Logan, Anne Dell, Brendan W. Wren

**Affiliations:** From the ‡ Department of Pathogen Molecular Biology, London School of Hygiene and Tropical Medicine, Keppel Street, London WC1E 7HT, United Kingdom,; the § Department of Life Sciences, Imperial College London, South Kensington Campus, London SW7 2AZ, United Kingdom,; the ¶ Institute of Child Health, University College London, 30 Guilford Street, London WC1N 1EH, United Kingdom,; the ** Vaccine Program, Human Health Therapeutics Portfolio, National Research Council, Ottawa, Ontario K1A 0R6, Canada, and; ‖ BioPharmaSpec Ltd., Suite 3.1, Lido Medical Centre, St. Saviours Road, Jersey JE2 7LA, United Kingdom

**Keywords:** bacteria, biofilm, glycosylation, glycosyltransferase, mutant, Clostridium difficile, flagella, gram positive bacteria

## Abstract

*Clostridium difficile* is the principal cause of nosocomial infectious diarrhea worldwide. The pathogen modifies its flagellin with either a type A or type B *O*-linked glycosylation system, which has a contributory role in pathogenesis. We study the functional role of glycosyltransferases modifying type B flagellin in the 023 and 027 hypervirulent *C. difficile* lineages by mutagenesis of five putative glycosyltransferases and biosynthetic genes. We reveal their roles in the biosynthesis of the flagellin glycan chain and demonstrate that flagellar post-translational modification affects motility and adhesion-related bacterial properties of these strains. We show that the glycosyltransferases 1 and 2 (GT1 and GT2) are responsible for the sequential addition of a GlcNAc and two rhamnoses, respectively, and that GT3 is associated with the incorporation of a novel sulfonated peptidyl-amido sugar moiety whose structure is reported in our accompanying paper (Bouché, L., Panico, M., Hitchen, P., Binet, D., Sastre, F., Faulds-Pain, A., Valiente, E., Vinogradov, E., Aubry, A., Fulton, K., Twine, S., Logan, S. M., Wren, B. W., Dell, A., and Morris, H. R. (2016) *J. Biol. Chem.* 291, 25439–25449). GT2 is also responsible for methylation of the rhamnoses. Whereas type B modification is not required for flagellar assembly, some mutations that result in truncation or abolition of the glycan reduce bacterial motility and promote autoaggregation and biofilm formation. The complete lack of flagellin modification also significantly reduces adhesion of *C. difficile* to Caco-2 intestinal epithelial cells but does not affect activation of human TLR5. Our study advances our understanding of the genes involved in flagellar glycosylation and their biological roles in emerging hypervirulent *C. difficile* strains.

## Introduction

*Clostridium difficile*, recently reclassified as *Peptoclostridium difficile* (1), has emerged since the mid-2000s as the main cause of antibiotic-associated diarrhea within healthcare environments (2). This Gram-positive spore-forming anaerobe is an opportunistic pathogen that colonizes the gastro-intestinal tracts of susceptible individuals, leading to *C. difficile* infection (3). Those at risk include patients undergoing antibiotic therapy, the immunocompromised, and the elderly (4), although increasingly younger people are affected by *C. difficile* infection (5).

Genetic typing and sequence analysis have confirmed that there are at least five distinct lineages of *C. difficile* (6, 7), which can be divided according to the most important PCR ribotypes (RTs)^4^: RT017, RT023, RT027, RT078, and a large group including the 630 strain and the other RTs (8, 9). Our work is focused on two lineages (RT023 and RT027). The lineage composed primarily of the RT027 strains has spread globally. It has also been associated with increased severity of disease and is described as “hypervirulent” (10–12). RT023 is a recently emerging lineage, prevalent in Europe, and is also often associated with more severe disease (13). Most strains of *C. difficile* produce two cytotoxins, toxin A (TcdA) and toxin B (TcdB), which cause extensive damage to the epithelial cells of the gastro-intestinal tract and are considered to be essential for *C. difficile* disease (14). The production of either TcdA or TcdB has been found to be essential for *C. difficile* infection (15, 16). However, the existence of naturally toxin A-negative strains suggests that other factors are involved in *C. difficile* transmissibility, survival, and colonization. *C. difficile* expresses multiple surface proteins and colonization factors, including cell surface-associated proteins (surface layer proteins), fibronectin-binding protein A, proteases, heat-shock proteins, and flagella (17–19). Flagella biosynthesis is known to be linked to a number of other cellular processes, including sporulation, adhesion, metabolism, and toxin production (20). The *C. difficile* flagella are composed of a single flagellin protein encoded by a single flagellin gene (*fliC*), which is called CD0239 in 630 strain and CDR20291_0240 in R20291 strain. *C. difficile* flagellin is post-translationally modified by an *O*-linked glycan, and this post-translational modification (PTM) is essential for motility (21). Two types of flagellin PTM have been identified in *C. difficile*, which we have previously defined as type A and B, where type A has a simpler structural modification of the flagellin than type B (21–23). The flagellin of the first *C. difficile* sequenced strain, 630, is modified at up to seven sites with a type A PTM (21–23), which is composed of an *O*-linked *N*-acetyl glucosamine (GlcNAc) linked to a methylated threonine via a phosphodiester bond (21–23). The genes responsible for this modification (CD0240, CD0241, CD0242, CD0243, and CD0244) are encoded immediately downstream of the flagellin gene, *fliC* (21–24). Previous genetic analysis of the hypervirulent RT027 strain revealed that the gene lying downstream of *fliC* in the type B modification, glycosyltransferase 1 (GT1 (CDR20291_0241)), was highly similar to CD0240 of 630 (21). Although the function of the GT1 protein is yet to be confirmed, the first sugar of the type B PTM is also an *O*-linked *N*-acetylhexosamine (HexNAc) residue, indicating that the GT1 protein may have a function similar to the CD0240 protein (22, 25). Beyond this initial sugar, however, the RT027 flagellin does not contain a phosphodiester-linked amino acid as in type A flagellin. Instead, it has recently been found to be extended by two rhamnoses and a novel sulfonated peptidyl amidoglycan (25).

The differences between type A and type B flagellin structures are reflected at the genetic level. In this study, bioinformatic analyses reveal the presence of two putative glycosyltransferase genes (GT2 (CDR20291_0242) and GT3 (CDR20291_0243)) in type B that are absent in the type A PTM gene cluster. Additionally, immediately downstream of GT2 and GT3, there are four further predicted coding sequences (CDR20291_0244-0247) that do not appear to code for glycosyltransferases. We also identified putative glycosylation genes upstream of *fliC* (CDR20291_0223-0226), which are similar to rhamnose biosynthesis genes. In addition, we explored which genes affect flagellin type B modification and investigated the biological role of flagellin glycosyltransferases from two hypervirulent *C. difficile* lineages, namely RT027 (strain R20291) and RT023 (strain CD1426). We show that flagellin PTM type B affects motility and adhesion properties, including adhesion to intestinal cells, but does not activate human TLR5.

## Results

### 

#### 

##### Flagella Glycosylation Genes in RT027 and RT023 Are Highly Similar

**FIGURE 1.**
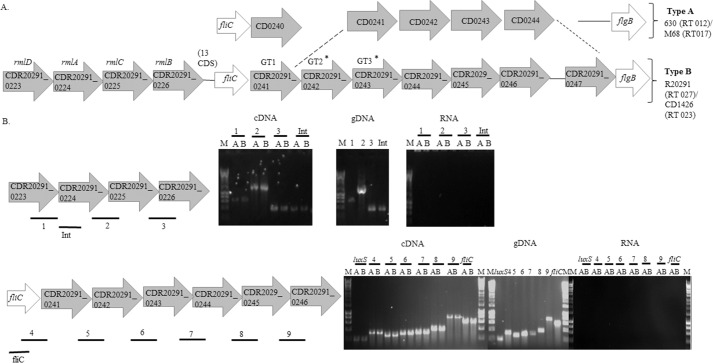
*A*, a schematic diagram illustrates flagellin type A (present in RT012 and RT017 strains) and type B (present in RT027 and RT023 strains) modification genes from *C. difficile*. Flagellar structural genes (*fliC* and *flgB*) are *colored* in *white*, and predicted GTs and remaining putative modification genes are *colored* in *gray*. The *dotted lines* indicate that there are homologous genes in PCR ribotypes 012/017 (CD0241–CD0244) and homologous genes in PCR ribotypes 023/027 (CDR20291_0242–0247). The *asterisk* indicates predicted GTs that are different in RT027 and RT023 compared with strain 630. CDR20291_0223–0226 are similar to *rmlD*, *rmlA*, *rmlC*, and *rmlB*, respectively. The number of coding sequences from CDR20291_R0226 and the *fliC* gene is also indicated. *B*, flagella glycosylation gene RT-PCR. *Top*, rhamnose biosynthetic genes. *Bars below* the genes illustrate the amplified regions (*1–3*) and internal region (*Int*) amplified as a control of gene expression by RT-PCR. *Bottom*, flagella glycosylation locus located downstream of the *fliC* gene. *Bars below* the genes illustrate the amplified regions (*4–9*). *luxS* gene amplification was used as a control for constitutive expression, and an internal primer was used to amplify *fliC* as a control of gene expression. Control reactions were carried out without reverse transcriptase (*No RT*) and with a DNA template from genomic DNA (*gDNA*). RT-PCR was carried out on RNA extracted during early exponential growth (*A*) and during late exponential growth (*B*).

We reported the presence of orthologues of the RT027 flagellin type B modification genes in RT023 strains. Thus, a BLAST analysis upstream and downstream of the RT027 flagellin biosynthetic locus identified glycosyltransferases and glycan biosynthesis genes (Fig. 1*A*). Downstream of the RT027 flagellin gene there were three putative glycosyltransferase genes (CDR20291_0241, CDR20291_0242, and CDR20291_0243, which we designate GT1, GT2, GT3, respectively) encoding predicted proteins with similarity to the group II superfamily of glycosyltransferases, each having a Pfam domain 00535 (26). In addition, GT2 also appears to have a methyltransferase domain (Pfam 08241). We identified four other predicted coding sequences (CDR20291_0244–0247) downstream of GT3 in the chromosome from strains representing RT027 and RT023, which may also be involved in this flagellin PTM (Fig. 1*A*). The CDR20291_0244 predicted protein shares some similarity with FemAB superfamily proteins, which contain an acyltransferase domain (Pfam 02388). CDR20291_0245 is a putative carbamoyl-phosphate synthetase with an ATP-grasp domain (Pfam 13535). This family includes a diverse set of enzymes that possess ATP-dependent carboxylate-amine ligase activity. CDR20291_0246 is a putative ornithine cyclodeaminase with a conserved domain PRK06046 also present in alanine dehydrogenase enzymes. CDR20291_0247 shows significant similarity to FdtB (dTDP-6-deoxy-d-xylohex-3-uloseaminase) from *Aneurinibacillus thermoaerophilus* and *Xanthamonas campestris*. In these organisms, this enzyme has been shown to be part of the biosynthetic pathway of TDP-d-Fuc*p*3NAc producing dTDP-d-Fuc*p*3N from dTDP-6-deoxy-d-xylohex-3-ulose (27, 28). Upstream of the flagellin biosynthesis genes, in both RT027 and RT023 strains, there were four genes (CDR20291_0223–0226) that are similar to rhamnose biosynthetic genes (*rmlD*, *rmlA*, *rmlC*, and *rmlB*, respectively) (Fig. 1*A*).

Additionally, none of the studied genes in Fig. 1*A* corresponds to a glycosulfotransferase that could potentially be involved in the biosynthesis of the sulfonated peptidylamido-sugar moiety.

To determine whether the putative flagellin type B modification genes upstream and downstream of *fliC* are co-transcribed with *fliC*, RT-PCR was carried out in R20291 (RT027) over the junctions between CDR20291_0223–0226 and GT1, GT2, GT3, and CDR20291_0244–0246. We found that the predicted type B modification genes were co-transcribed in both exponential and stationary growth phase (Fig. 1*B*).

##### Mutations in Flagellin Modification Type B Genes Result in Truncation or Abolition of Flagellin Post-translational Modification

**FIGURE 2.**
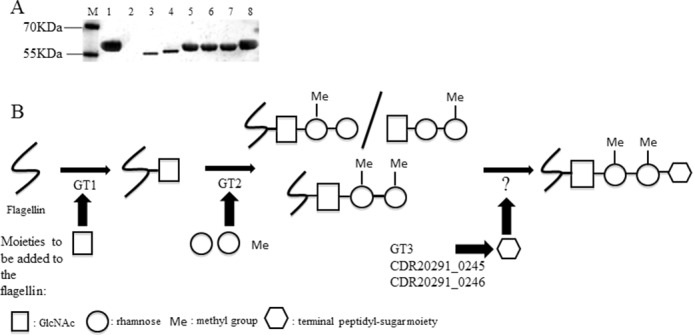
*A*, Western blotting of flagellin extractions of RT027 wild type and flagellin modification mutants. *Lane M*, marker (prestained protein marker; Fermentas); *lane 1*, R20291; *lane 2*, R20291*fliC; lane 3*, GT1; *lane 4*, GT2; *lane 5*, GT3; *lane 6*, CDR20291_0245; *lane 7*, CDR20291_0246; *lane 8*, GT1comp. *B*, schematic diagram illustrates the structure of flagellin type B modification and the contribution of each GT. Flagellin type B is modified with one *N*-acetylglucosamine (*white square*) linked to two rhamnoses (*white circles*) (with or without a methyl group (*Me*)). *White hexagon*, terminal peptidyl-sugar moiety.

Disruption of the glycosyltransferase gene CD0240, as well as CD0241, CD0242, CD0243, CD0244 in 630 strain was previously reported to result in flagellin of different sizes (23). To further characterize the role of type B modification genes in flagellin glycosylation, R20291 mutants in GT1, GT2, GT3, CDR20291_0245, and CDR20291_0246 were generated by ClosTron mutagenesis. Flagellin samples were studied by SDS-PAGE and mass spectrometry. On SDS-PAGE, flagellin of R20291 had a molecular mass around 60 kDa (Fig. 2*A*). We also observed a shift in the mass of the flagellin of all flagella glycosylation mutants, especially in GT1 and GT2, toward lower molecular weight. Wild type molecular weight was restored upon complementation in *trans* of GT1 mutant (GT1comp) (Fig. 2*A*).

Gel bands corresponding to flagellin of mutants and corresponding complements were analyzed by nano-LC-MS/MS. Spectra were manually compared, and the identified glycopeptides in the mutants are listed in Table 1. Tandem mass spectrometric analyses of flagellin tryptic digests from the GT1 mutant showed peptides to be non-glycosylated. Flagellin isolated from the GT2 mutant harbored glycopeptides with a single HexNAc moiety but lacking any further extension of the glycan. Flagellin isolated from the GT3 mutant was modified by a deoxyHex-deoxyHex-HexNAc (with and without methyl groups) but lacking the terminal sulfonated peptidylamido-sugar moiety observed in the wild type (25). Similarly, the CDR20291_0245 and CDR20291_0246 mutants carried the non-extended core trisaccharide (Table 1). The analysis of the complemented mutants by mass spectrometry demonstrated that the flagellar glycan chain is fully restored (data not shown).

**TABLE 1:**
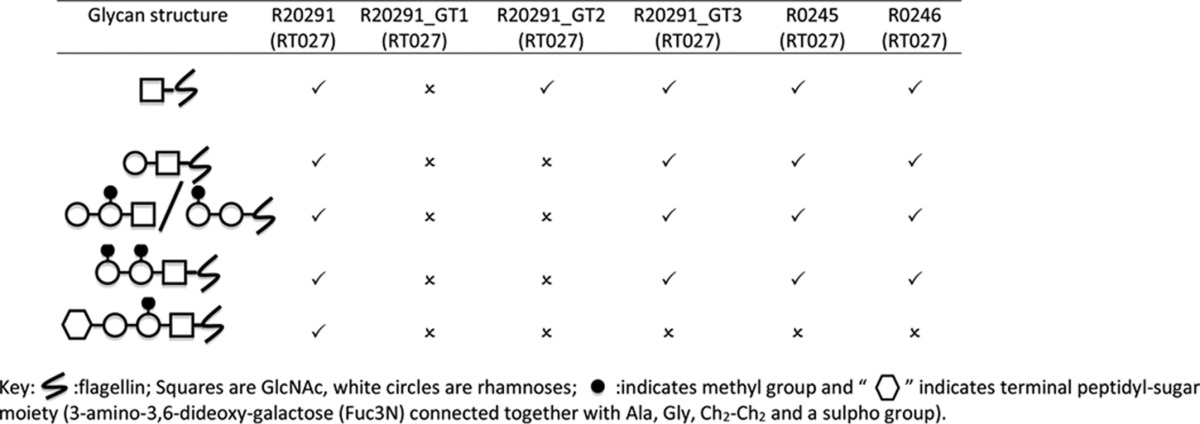
**Glycan structures identified by mass spectrometry present on *C. difficile* flagellin purified from mutant strains belonging to ribotypes 027 and 023** A check mark indicates presence, and an X indicates absence. The glycan structures of wild-type of both RT027 and RT023 have been described in the accompanying article (25) and are shown as a reference.

Based on the mass spectrometric data of the mutants and taking into account the flagellin type B sulfonated peptidylamido-glycan structure reported by Bouché *et al.* (25), we conclude that the glycosyltransferases GT1 and GT2 are responsible for the sequential addition of the GlcNAc and two rhamnoses sugars, respectively, and that GT3, CDR20291_0245, and CDR20291_0246 are all involved in the biosynthesis of the terminal sulfonated peptidylamido-sugar moiety. Given that our bioinformatics analysis suggests that GT2 could also function as a methyltransferase, we hypothesize that GT2 is additionally responsible for methylation of the rhamnoses (Table 1 and Fig. 2*B*).

##### Motility, Cell Aggregation, and Biofilm Formation Are Affected by Flagellin Glycosylation Mutants in RT027 and RT023 Strains

**FIGURE 3.**
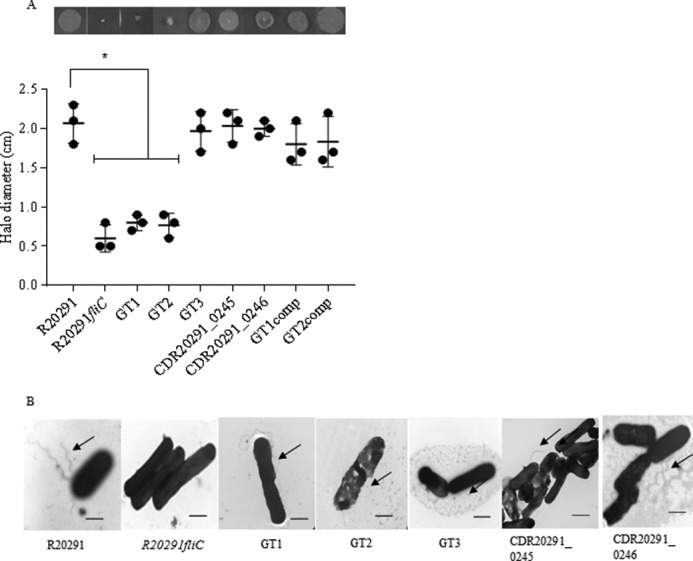
**Motility and flagella production in flagellin modification mutants.** *A*, quantification of the motility halo of RT027 WT (R20291) and corresponding mutants (*fliC*, GT1, GT2, GT3, R20291_0245, and R20291_0246) and complements (GT1comp and GT2comp). Measurements are in cm. *Asterisks*, significant differences compared with WTs (*p* < 0.05). Data are presented as the mean ± S.E. (*error bars*), *n* = 3. *B*, electron transmission images of R20291 and its corresponding flagellin modification mutants. *Arrows*, flagella; *bar*, 0.5 nm.

Flagella type A modification is essential for motility in *C. difficile* 630 and RT017 strains (23). To determine whether flagellin modification type B is also required for motility, GT1, GT2, GT3, CDR20291_0245, and CDR20291_0246 mutants were assessed. The R20291*fliC* mutant was used as a negative control. Measuring bacterial motility in agar, we found that GT1 and GT2 knock-out strains were significantly less motile than wild type R20291 (diameter of 0.8 ± 0.1 cm *versus* 2.0 ± 0.2 cm) (Fig. 3*A*). Complementation of these knockouts restored the motility phenotype (Fig. 3*A*). Furthermore, GT3, CDR20291_0245, and CDR20291_0246 mutants were as motile as the parent R20291 strain, suggesting that the first three sugars of the flagella glycan chain are both sufficient and necessary for full motility of *C. difficile*.

To study the effect of PTM type B on flagellar assembly, individual bacteria were imaged by TEM. The images revealed the presence of flagella filaments associated with the surface of R20291 and mutants. The R20291*fliC* mutant was used as a negative control (Fig. 3*B*). In addition, we quantified flagella filament length of R20291 and its corresponding mutants, and no significant differences were observed (Table 2). Our results suggest that type B modification is not required for flagella assembly.

**TABLE 2:** **Measurement of flagella filament length**

Strains	Length of flagella filament ± S.E.*^a^*
	μ*m*
R20291	3.62 ± 0.42
GT1	3.55 ± 0.46
GT2	3.60 ± 0.40
GT3	3.63 ± 0.33
CDR20291_0245	3.66 ± 0.45
CDR20291_0246	3.64 ± 0.42

*^a^* The flagella of 100 bacteria were measured in each strain, and TEM images were analyzed using the line measuring tool of the Image J 1.50i software (National Institutes of Health).

**FIGURE 4.**
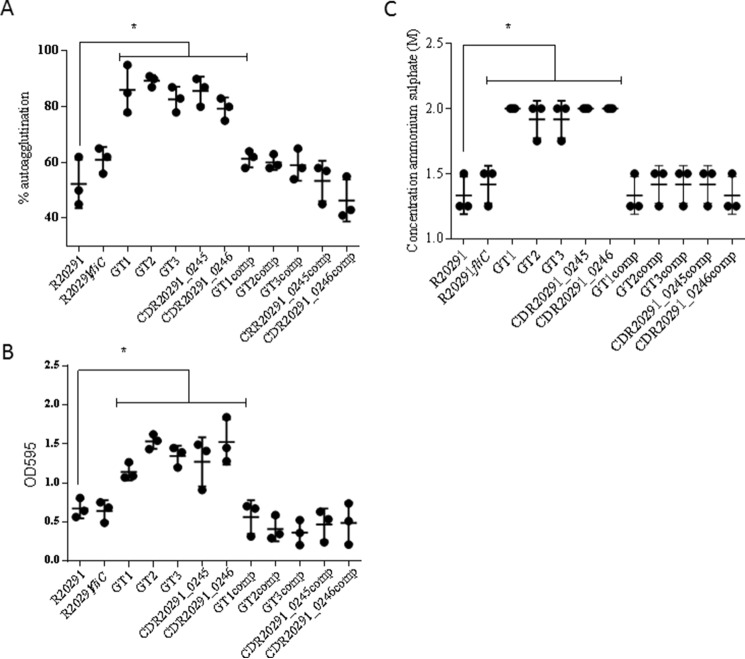
**Cell aggregation in PBS (*A*), biofilm formation (*B*), and cell hydrophobicity (*C*).** All of the type B modification mutants showed a significant increase in cell aggregation, biofilm formation, and hydrophobicity compared with R20291. *Asterisks*, significant differences compared with WT (*p* < 0.05). Data are presented as the mean ± S.E. (*error bars*) from three independent experiments with three technical replicates each.

We next investigated whether flagellin PTM affects cell aggregation of hypervirulent *C. difficile* RT027 and observed increased aggregation in all glycosylation mutants (83 ± 3 μm) *versus* wild type (R20291) (52 ± 5 μm) (Fig. 4*A*). These results indicate that flagella post-translational modification type B affects *C. difficile* cell aggregation. However, a FliC mutant strain that completely lacks flagellin aggregates similarly to R20291, suggesting that flagella *per se* are not required for cell aggregation. Complementation of the mutants restored the original aggregation phenotype (Fig. 4*A*). Cell surface hydrophobicity of wild type and flagellin glycosylation mutants was measured, and our results show that flagellin PTM mutants are more hydrophobic than the wild-type and/or R20291*fliC* (Fig. 4*C*). Cell viability in PBS1x was affected after 16-h incubation (Table 3) but in the same way in the wild types (R20291 and CD1426) and corresponding mutants. Cell aggregation was visualized by optical microscopy, where mutants showed a clear tendency to cell-to-cell aggregate (supplemental Fig. 1).

**TABLE 3:** ***C. difficile* viability in PBS1x** cfu/ml were counted when the experiment was set up (initial cfu/ml) and after 16 h of incubation (final cfu/ml).

Strains	Initial cfu/ml ± S.E.	Final cfu/ml ± S.E.
R20291	2.3 ± 0.2E+09	1.3 ± 0.3E+07
GT1	2.1 ± 0.4E+09	1.0 ± 0.3E+07
GT2	2.5 ± 0.3E+09	1.5 ± 0.1E+07
GT3	2.4 ± 0.3E+09	1.3 ± 0.3E+07
CDR20291_0245	2.2 ± 0.1E+09	1.4 ± 0.2E+07
CDR20291_0246	2.2 ± 0.3E+09	1.9 ± 0.5E+07
GT1comp	2.6 ± 0.2E+09	1.3 ± 0.2E+07
GT2comp	2.7 ± 0.2E+09	1.5 ± 0.3E+07
GT3comp	2.2 ± 0.4E+09	1.1 ± 0.3E+07
CDR20291_0245 comp	2.3 ± 0.2E+09	1.3 ± 0.1E+07
CDR20291_0246 comp	2.2 ± 0.4E+09	1.5 ± 0.3E+07
CD1426	2.1 ± 0.2E+09	1.6 ± 0.1E+07
CD1426_GT2	2.2 ± 0.3E+09	1.2 ± 0.2E+07
CD1426_GT2comp	2.4 ± 0.5E+09	1.5 ± 0.3E+07

To determine whether these observations can be extended to another strain from a different lineage, we repeated these experiments using the GT2 mutant in CD1426 (RT023). Analogous to R20291 (RT027), this mutant (supplemental Fig. 2*A*) was less motile compared with the wild type strain (diameter of 0.8 ± 0.1 cm *versus* 2.1 ± 0.1 cm) and aggregated more than the CD1426 mutant (85 ± 1 *versus* 60 ± 3, respectively) (supplemental Fig. 2*B*).

Finally, we determined the role of flagellin modification in RT027 and RT023 on biofilm on abiotic surface (polystyrene plates). As shown in Fig. 4*B*, flagellin type B modification facilitates adherence of cells to abiotic surfaces when compared with the wild type. Complementation of the mutants restored the original phenotype.

##### C. difficile Flagellin Glycosylation Modulates Bacterial Adherence to Intestinal Epithelial Cells

**FIGURE 5.**
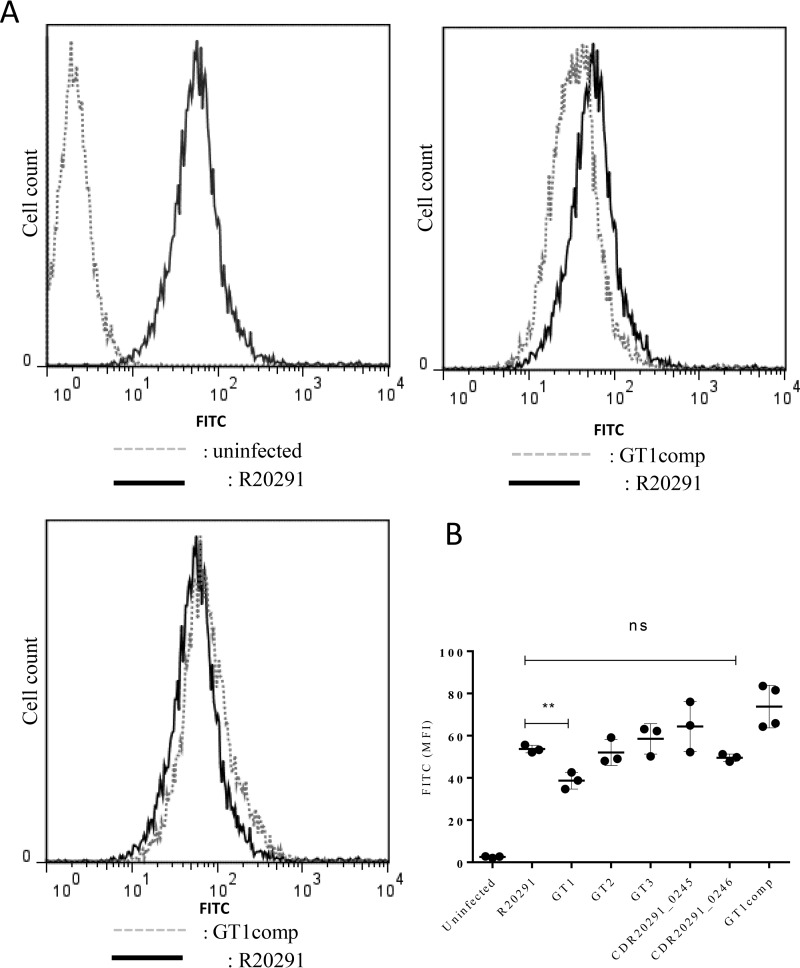
**Cell adhesion in RT027 flagellin modification mutants.** *C. difficile* flagella glycosylation moieties contribute to bacterial adherence to human IECs. 1 × 10^6^ Caco-2 cells were infected with 2.5 × 10^7^ FITC-labeled WT R20291 and flagellin glycosylation isogenic mutants and co-cultured for 1.5 h under anaerobic conditions. Bacterial adherence was assessed by flow cytometry (*A*) and quantified as change in median fluorescence intensity (*B*). Data are presented as the mean ± S.E. (*error bars*) from three independent experiments with three technical replicates each. **, *p* < 0.01; *ns*, non-significant.

Having observed differences in the glycosylation mutants in aggregation and biofilm on an abiotic surface, we investigated whether adhesion to a physiologically relevant biological surface, such as human intestinal epithelial cells (IECs), may also be affected. Previous work has shown that the *C. difficile* R20291*fliC* mutant adheres less well to human IECs than the wild type strain (29), but whether adhesion is influenced by changes in flagellin glycosylation is unknown. Therefore, we measured adherence of FITC-labeled R20291 *C. difficile*, as well as the respective flagellin type B modification mutants, to cultured Caco-2 cells by flow cytometry (Fig. 5*A*). Compared with R20291, only the GT1 mutant showed reduced adherence, and complementation restored the phenotype (Fig. 5*B*). All other mutants adhered similarly to R20291 on the tested cell line. These data suggest that flagellin glycans facilitate adhesion of *C. difficile* to IECs and that the presence of just the first sugar in the glycan chain is sufficient to restore adhesion to wild type levels.

Similarly, the CD1426 GT2 mutant showed no difference in adherence compared with the WT strain, confirming that GT2 does not affect bacterial adherence to IECs (data not shown). Of note, no difference in FITC labeling was observed between WT and glycosylation mutants (data not shown).

##### C. difficile Flagellar Glycosylation Is Not Involved in TLR5 Recognition

**FIGURE 6.**
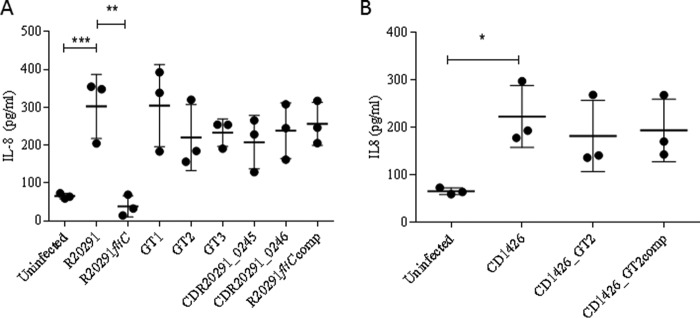
**TLR5 activation in RT027 and corresponding mutants.** Glycosylation moieties on *C. difficile* flagellin do not contribute to TLR5 activation. Untransfected (HEK-WT) and TLR5-transfected (HEK-TLR5) HEK cells were co-cultured with WT R20291 (*A*) or CD1426 (*B*) and their respective flagellin mutants at a multiplicity of infection of 10. IL-8 protein was quantified 8 h post-infection. Data are presented as the mean ± S.E. from three independent experiments with three technical replicates each. *, *p* < 0.05; **, *p* < 0.01; ***, *p* < 0.001.

TLR5 is part of the human innate immune system and specifically binds to bacterial flagellin. TLR5 activation results in the secretion of the cytokine IL-8. The potential impact of flagellin glycosylation (*e.g.* masking) on host TLR5 engagement was investigated. Untransfected and TLR5-transfected human HEK293 cells were co-cultured with wild type *C. difficile* RT027 and RT023 strains and their respective flagella modification-deficient mutants. 8 h after infection, IL-8 secretion into the medium was quantified by ELISA. Minimal IL-8 was recorded in untransfected cells; in contrast, significant increase in IL-8 was observed in response to the wild type R20291 (Fig. 6*A*). The increase was completely abrogated in response to the R20291*fliC* mutant, whereas the flagella glycosylation mutants activated TLR5 to a similar extent as the WT strain (Fig. 6*A*). The CD1426 glycosylation mutant also displayed similar TLR5 engagement as its WT counterpart (Fig. 6*B*). These observations suggest that the interaction between TLR5 and *C. difficile* flagellin is maintained even in the absence of glycans and therefore that glycosylation of flagellin in *C. difficile* does not influence TLR5 binding or activation.

## Discussion

*C. difficile* is a major global healthcare problem. In recent years, hypervirulent strains, including RT027 and RT023, have emerged, causing more severe infections (29, 30). Although the toxins are the main virulence factors, other features, such as flagella (31), S-layer protein (32), and sporulation (33), also contribute to bacteria colonization and transmission of the disease.

*C. difficile* flagellin (FliC) has a role in motility, colonization, biofilm formation, and toxin production. Moreover, flagellin is immunogenic and protective in a murine model of *C. difficile* infection (34). However, little is known about the role of protein PTM in *C. difficile* disease, which has a type A or type B modification (23, 25). It has also been reported that type A post-translational modification of flagellin in *C. difficile* 630 strain has a contributory effect in pathogenesis (23). However, the role of type B post-translational modification of flagellin remains unclear. In this work, we investigated the function of putative type B flagellin modification genes and demonstrated the importance of flagellar modification in motility, cell aggregation, biofilm formation, epithelial cell adhesion, and TLR5 recognition.

To study the function of the flagellin type B modification genes, we generated mutants and studied the consequences for flagellin post-translational modification using nano-LC-MS/MS. Based on our results and knowledge of type B glycosylation (25), we demonstrated that the flagellin type B PTM locus contains two sequential glycosyltransferase genes (GT1 and GT2) lying downstream of the *fliC* gene. Our data suggest that GT1 is responsible for GlcNAc addition to the flagellin polypeptide, whereas GT2 appears to be responsible for the extension of this *O*-linked monosaccharide with an additional two rhamnose moieties. Bioinformatic analysis showed that GT2 has a methyltransferase domain (Pfam 08241). We propose that GT2 is responsible for both the addition and the monomethylation of these rhamnose residues. Bifunctional glycosyltransferases have also been reported in other human pathogens, such as *Listeria monocytogenes* (35). Interestingly, we found that methylation of the second rhamnose only occurred in mutants that were truncated at the trisaccharide, and not in the WT, suggesting that methylation of this sugar may prevent further extension of the glycan. Three mutants were found to carry glycans that were not extended from the trisaccharide and are therefore predicted to be involved in the biosynthesis of the unusual sulfonated peptidylamido-sugar moiety that is found in the WT (25). These were the putative glycosyltransferase GT3, the putative carbamoyl-phosphate synthetase CDR20291_0245, and the putative ornithine cyclodeaminase CDR20291_R0246. Although it is clear that these enzymes are involved in the flagellin post-translational modification, their precise roles remain to be determined. Despite several attempts, we were unsuccessful in creating mutants for the two remaining genes in the PTM locus, namely CDR20291_0244 and CDR20291_0247. These genes are located on either side of CDR20291_0245 and CDR20291_0246, downstream from GT1, GT2, and GT3. Our failure to produce mutants strongly suggests that both genes are important for the viability of *C. difficile* and may be involved in essential metabolic pathways.

In many bacteria, such as *C. jejuni*, the genes coding for the biosynthesis of the glycan and the corresponding glycosyltransferases are located adjacent to the biosynthetic flagellar genes (36). For type B flagellin, it appears that the rhamnose biosynthetic group of genes (CDR20291_0223–0226), similar to *rmlD*, *rmlA*, *rmlC*, and *rmlB*, lie upstream of *fliC* and are co-transcribed with both the *fliC* gene and the downstream GT loci. Our repeated efforts to create mutants in the rhamnose genes were unsuccessful, which could suggest that these genes would be involved in metabolic pathways that are essential for *C. difficile* viability.

In some bacterial pathogens, flagellin PTM is essential for bacterial flagellar assembly (37), although in *Pseudomonas* spp. and *Burkholderia* spp., glycosylation is not required for flagellar assembly (38). Type B flagellin PTM in *C. difficile* is not required for flagellar assembly because flagellar GT mutants still produce flagella. This is similar to type A flagellar modification, where glycosylation was also not required for flagellar filament assembly (21, 23). Several bacterial pathogens, such as *Pseudomonas aeruginosa*, decorate their flagellin with sugars that are not required for motility (39). However, in most of the bacterial pathogens, flagellar glycosylation does affect motility (23, 40). The latter was previously shown to be the case for *C. difficile* 630, which produces a type A modification (21, 23). Our current study demonstrates that the flagellin PTM type B similarly facilitates the motility of *C. difficile* RT027 and RT023 strains, possibly by altering the physical properties of the cell.

Flagellin glycosylation can also alter adhesion properties of the bacterial cells (23). Our results suggest that flagellin PTM type B has an impact on biofilm formation on abiotic surfaces. Additionally, flagellin type B glycosylation affects adhesion to Caco-2 cells, which is a cell model widely used for studying *C. difficile* cell adhesion, but it has also its limitations due to an absence of mucus layer. Further studies on a mucus-producing cell line could give us a better understanding of the interaction between *C. difficile* flagellin glycan and the intestinal tissue.

Cell autoaggregation is a phenomenon observed as the clumping of cells. The importance of aggregation in virulence has been strongly implicated for other pathogenic bacteria, including *Yersinia enterocolitica*, *Vibrio cholerae*, and *Campylobacter jejuni*. In those cases, a defect in cell aggregation showed a defective colonization phenotype in the corresponding animal model (41–43). However, in *C. difficile* 630 strain, an increase in cell aggregation attenuates mouse colonization (23). Our results indicate that flagellin modification type B affects *C. difficile* cell aggregation and biofilm formation. We also showed that differences in flagellin modification also alter bacterial cell hydrophobicity, causing increased aggregation of the cells, which could prevent them from swimming efficiently. This phenotype might affect the fitness of the bacteria in the host and also interfere with the immune system. Although the flagellum is important for bacterial motility, cell aggregation, and colonization, little is known about the functional role of flagellar glycosylation in host-bacteria interactions. Activation of TLR5 by flagellin initiates a powerful host response that provides essential signals for maintaining intestinal immune homeostasis (44). In *C. difficile*, it has been described that the flagellin protein activates TLR5 in epithelial cells (45), but little is known about the interaction between flagellin glycosylation and the immune response in *C. difficile*. In *P. aeruginosa* and *Burkholderia cenocepacia* flagellin, post-translational modification modulates innate immune responses in human epithelial cells (46). In the current study, we confirmed that *C. difficile* flagellin is required for activation of TLR5, but glycosylation deficiency did not affect TLR5 signaling. Our data do not, however, preclude the possibility that the *C. difficile* flagellin sulfonated peptidylamido-glycans alter the host immune response in other ways. For example, it has recently been reported that pseudaminic acid on *C. jejuni* flagellin interacts with the host in a TLR5-independent manner by engaging with a sialic acid binding lectin, consequently promoting anti-inflammatory immunity (47).

Our study provides clear evidence that the emerging hypervirulent *C. difficile* RT027 and RT023 strains possess at least two sequential glycosyltransferases (GT1 and GT2), which add GlcNAc, methyl-rhamnoses, and/or rhamnoses to flagellin, and highlights the importance of flagellin type B modification in motility, cell adhesion, and TLR5 engagement.

## Experimental Procedures

### 

#### 

##### Bacterial Growth and Culture

*C. difficile* was routinely grown on Brazier's CCEY agar (BioConnection, Leeds, UK) containing 4% (w/v) egg yolk, 250 mg/ml cycloserine, 8 mg/ml cefoxitin (Bioconnections), and 1% defibrinated horse blood (TCS Biosciences, Buckingham, UK), in blood agar (Oxoid, Hampshire, UK) and in BHI (brain heart infusion medium (Oxoid)) and BHIS (BHI supplemented with 0.5% (w/v) yeast (Sigma, Gillingham, UK), 0.1% l-cysteine (Sigma)) broth or agar. All cultures were grown from glycerol stocks in an anaerobic atmosphere (10% CO_2_, 10% H_2_, 80% N_2_) in a Don Whitney MG500 anaerobic workstation (Don Whitney Scientific Ltd.) at 37 °C. All *Escherichia coli* strains were grown using Luria-Bertani broth or agar supplemented with 12.5 mg/ml chloramphenicol (Sigma) at 37 °C. Plasmid DNA was transferred into *C. difficile* R20291 and CD1426 by conjugation from CA434 *E. coli*, as described previously (48, 49). Strains and plasmids, used in this study, are shown in Table 4.

**TABLE 4:** **Strains and plasmids used in this study**

Strains and plasmids	Characteristics	Source
**Strains**		
*C. difficile*		
R20291	Hypervirulent PCR ribotype 027, isolated from an outbreak in 2004–2005	Ref. 24
R20291*fliC*::CT	*fliC* mutant derived from R20291 by ClosTron insertion	This study
GT1::CT	GT1 mutant derived from R20291 by ClosTron insertion	This study
GT2::CT	GT2 mutant derived from R20291 by ClosTron insertion	This study
GT3::CT	GT3 mutant derived from R20291 by ClosTron insertion	This study
CDR20291_0245::CT	CDR20291_0245 mutant derived from R20291 by ClosTron insertion	This study
CDR20291_0246::CT	CDR20291_0246 mutant derived from R20291 by ClosTron insertion	This study
R20291*fliC*:: CT::*fliC*	R20291*fliC* mutant derived from R20291 containing the pMTL-*fliC* plasmid	This study
GT1::CT::GT1 (GT1comp)	GT1 mutant derived from R20291 containing the pMTL-GT1 plasmid	This study
GT2::CT::GT2 (GT2comp)	GT2 mutant derived from R20291 containing the pMTL-GT2 plasmid	This study
GT3::CT::GT3 (GT3comp)	GT3 mutant derived from R20291 containing the pMTL-GT3 plasmid	This study
CDR20291_0245::CT::R0245 (CDR20291_0245comp)	ORF5 mutant derived from R20291 containing the pMTL-CDR20291_0245 plasmid	This study
CDR20291_0246::CT::R0246 (CDR202911_0246comp)	ORF6 mutant derived from R20291 containing the pMTL-CDR20291_0246 plasmid	This study
CD1426	Hypervirulent PCR ribotype 023, isolated from an outbreak in 2010	Queens Hospital Remford
CD1426_GT2::CT	GT2 mutant derived from CD1426 by ClosTron insertion	This study
CD1426_GT2::CT::GT2 (CD1426_GT2comp)	GT2 mutant derived from CD1426 containing the pMTL-GT2 plasmid	This study
*E. coli*		
Top10	F− mcrA Δ(mrr-hsdRMS-mcrBC) ϕ80lacZΔM15 ΔlacX74 nupG recA1 araD139 Δ(ara-leu)7697 galE15 galK16 rpsL(Str^R^) endA1 λ^−^	Invitrogen
CA434	*E. coli* HB101 [F^−^ *mcrB mrr hsdS20*(r_B_^−^ m_B_^−^) *recA13 leuB6 ara-14 proA2 lacY1 galK2 xyl-5 mtl-1 rpsL20*(Sm^r^) *glnV44* λ^−^] containing plasmid R702	Ref. 50

**Plasmids**		
pMLT007C-E2-R20291*fliC*	ClosTron plasmid retargeted to *fliC* at 344/345a	DNA 2.0
pMLT007C-E2-GT1	ClosTron plasmid retargeted to GT1 at 478/479s	DNA 2.0
pMLT007C-E2-GT2	ClosTron plasmid retargeted to GT2 at 1293/1294s	DNA 2.0
pMLT007C-E2-GT3	ClosTron plasmid retargeted to GT3 at 1800/1801s	DNA 2.0
pMTL007C-E2-CDR20291_0245	ClosTron plasmid retargeted to CDR20291_0245 at 147/148s	DNA 2.0
pMTL007C-E2-CDR20291_0246	ClosTron plasmid retargeted to CDR20291_0246 at 392/393a	DNA 2.0
pMTL84153	*E. coli*–*C. difficile* shuttle plasmid (pCD6;*catP*; ColE1+*tra*; *fdx* promoter)	Ref. 51
pMTL-*fliC*	pMTL84151 with *fliC* cloned with its native promoter	This study
pMTL-GT1	pMTL84153 with GT1 cloned behind the *fdx* promoter	This study
pMTL-GT2	pMTL84153 with GT2 and GT3 cloned behind the *fdx* promoter	This study
pMTL-GT3	pMTL84153 with GT3 cloned behind the *fdx* promoter	This study
pMTL-CDR20291_0245	pMTL84153 with CDR20291_0245 cloned behind the *fdx* promoter	This study
pMTL-CDR20291_0246	pMTL84153 with CDR20291_0246 cloned behind the *fdx* promoter	This study

Growth curves were performed using BHIS medium, and samples were incubated at 37 °C in an anaerobic atmosphere. Samples were taken at different time points during the first 24 h. Experiments were performed in three separate biological replicates, measured as three technical replicates each.

##### Mutagenesis and Complementation Method

Knockouts of post-translational flagellin type B genes were constructed by insertional inactivation of the target genes with the ClosTron system as described previously (49). Primers are shown in supplemental Table 1. The retargeted plasmids were designed using the Perutka algorithm at the ClosTron website and synthesized by DNA 2.0. They were transferred from *E. coli* CA434 to wild-type R20291 and CD1426 by conjugation, and transconjugants were selected for on BHIS agar containing 15 μg/ml thiamphenicol (Sigma). Chromosomal insertion of the intron was selected for on BHIS agar containing 20–40 μg/ml lincomycin (Sigma) in R20291 and with 5 μg/ml erythromycin (Sigma) in CD1426. Mutants were checked by PCR and genome sequencing.

For complementation studies, genes were amplified by PCR using primer pairs (supplemental Table 1) and cloned into plasmid pMTL84153 to generate the plasmids listed in Table 4. Each of the constructs was transferred from *E. coli* CA434 into *C. difficile* mutants by conjugation, and transformants were selected on BHIS plates containing 15 μg/ml thiamphenicol to select for *C. difficile* containing the retargeted plasmid integrants.

##### RNA Isolation and RT-PCR

Bacteria were grown in 10 ml of BHIS overnight at 37 °C. The cell pellet was lysed using the FASTRNA Pro Blue kit (MP Biomedicals) according to the manufacturer's instructions for lysis of Gram-positives. Total RNA was extracted using the Qiagen RNeasy kit, as described in the manufacturer's instructions. The integrity and concentration of extracted RNA were determined using a Nanodrop spectrophotometer. RT-PCRs were carried out with random primers and Superscript II reverse transcriptase (Invitrogen) according to the manufacturer's instructions, using 1 μg of total RNA. The cDNA was used as amplicon to perform further PCRs. The PCR program was the following: 94 °C for 2 min; 35 cycles of denaturation (94 °C for 30 s); annealing (50–60 °C for 30 s); 72 °C for 3 min; and one extension cycle (72 °C for 5 min). The negative control without reverse transcriptase was carried out in 5 μl of diethylpyrocarbonate-treated water. Primers used are listed in supplemental Table 1. *luxS* gene amplification was used as a control for constitutive expression, and an internal primer was used to amplify *fliC* as a control of gene expression. We performed experiments in three separate biological replicates, measured as two technical replicates each.

##### Flagellin Extraction and Western Blotting

Liquid cultures were grown on BHIS broth overnight, at 37 °C. *C. difficile* strains were harvested, washed in phosphate-buffered saline, and resuspended in a 1:100 volume of low pH glycine (0.2 m glycine-HCl, pH 2.2) and incubated at room temperature for 30 min with gentle shaking. The cell pellets were removed by centrifugation at 4 °C, and the supernatant was neutralized with the addition of 2 m Tris to a neutral pH. Flagellin samples were normalized according to the *A*_600_ of the cultures, and a total volume of 10 μl was run on a Nu-Page 4–12% BisTris SDS-polyacrylamide gel in MOPS running buffer (both from Life Technologies, Inc.) for 1.5 h. Proteins were transferred to a nitrocellulose membrane using the iBLOT system (Life Technologies). The membrane was blocked in PBS plus 2% milk and probed with an anti-FliC antibody (a generous gift from the Armstrong laboratory, University of Calgary, Alberta, Canada) in PBS plus 0.1% skimmed milk and 0.1% Tween 20 at a 1:10,000 dilution for 1 h at room temperature. Following standard washing with PBS plus 0.1% Tween 20, a donkey anti-chicken IR dye 680RD secondary antibody (LI-COR) was added at 1:5000 in PBS plus 0.1% skimmed milk, 0.01% Tween 20, and 0.01% SDS and incubated in the dark for 1 h. The membrane was washed as before and visualized using a LI-COR Odyssey system.

##### Nano-LC-MS/MS Analysis

In preparation for mass spectrometry, flagellin samples were digested with trypsin (EC 3.4.21.4; Promega) overnight. Tryptic digests were analyzed by nano-LC-MS/MS using a reverse-phase nano-HPLC system (Dionex, Sunnyvale, CA) connected to a quadrupole TOF mass spectrometer (Q-STAR Pulsar I, MDS Sciex). The digests were separated by a binary nano-HPLC gradient generated by an Ultimate pump fitted with a Famos autosampler and a Switchos microcolumn switching module (LC Packings, Amsterdam, The Netherlands). An analytical C_18_ nanocapillary (75-meter inside diameter × 15 cm; PepMap) and a micro-precolumn C_18_ cartridge were employed for on-line peptide separation. The digest was first loaded onto the precolumn and eluted with 0.1% formic acid (Sigma) in water (HPLC grade; Purite) for 4 min. The eluant was then transferred onto an analytical C_18_ nanocapillary HPLC column and eluted at a flow rate of 150 nl/min using the following gradient of solvent A (0.05% (v/v) formic acid in a 95:5 (v/v) water/acetonitrile mixture) and solvent B (0.04% formic acid in a 95:5 (v/v) acetonitrile/water mixture): 99% A from 0 to 5 min, 99 to 90% A from 5 to 10 min, 90 to 60% A from 10 to 70 min, 60 to 50% A from 70 to 71 min, 50 to 5% A from 71 to 75 min, 5% A from 75 to 85 min, 5 to 95% A from 85 to 86 min, and 95% A from 86 to 90 min. Data acquisition was performed using Analyst QS software with an automatic information-dependent acquisition function.

##### Motility and EM

Bacteria were grown on BHIS plates for 24 h at 37 °C. One colony was inoculated in BHIS with 0.3% agar (BD Bacto-agar) and incubated in the anaerobic hood at 37 °C for 48 h. Photographs were captured after the incubation time with a Canon 600D SLR camera. The diameter of the halo was also measured. We performed experiments in three separate biological replicates, measured as three technical replicates each.

For *C. difficile* visualization by EM, strains were grown in BHIS tubes at 37 °C overnight under anaerobic conditions. Each strain was grown in a total volume of 1 ml and was incubated for 24 h. 500 μl of 2.5% paraformaldehyde, 2.5% glutaraldehyde, 0.1 m sodium cacodylate, pH 7.4, was added to 500 μl of the bacterial culture. Five microliters of each sample were added to 200 μl of deionized water (Sigma). Five microliters were placed onto a platform-coated 300 mesh copper grid for 1 min. The sample was then stained with 10 μl of 0.3% phosphotungstic acid, pH 7, for 1 min. The phosphotungstic acid was drained, and the grid was air-dried before examining on the Jeol 1200EX transmission electron microscope. Digital images were recorded using a side-mounted AMT 2K CCD Digital camera supplied by Deben UK Ltd., IP30 9QS.

##### Cell Aggregation and Hydrophobicity Assays

The cell aggregation assay was performed according to Faulds-Pain *et al.* (23). Briefly, *C. difficile* strains were grown on BHIS agar for 24 h and suspended in PBS to obtain an *A*_600 nm_ of 10.0 ± 0.1 in a volume of 5 ml. Following incubation for 16 h at 37 °C in the anaerobic cabinet, the top 1 ml was removed, and the *A*_600 nm_ was measured. The *A*_600 nm_ of the whole solution was also measured. Cell aggregation was calculated as the difference between the *A*_600 nm_ of the whole solution and the *A*_600 nm_ of the top 1 ml as a percentage of the whole solution OD. Bacterial cell suspensions in 1× PBS were fixed with cold methanol 100% and stained with 1% crystal violet for 1 min. After washing with distilled water, bacterial cells were mounted on glass slides using PBS/glycerol (1:1). Imaging was performed with a Leica microscope (Leith DMRB), Qimaging Retina 2000R FAST1394 camera, and Volocity software (PerkinElmer Life Sciences).

The mutants and their corresponding complements were compared with the wild type. Cell viability of bacterial suspensions in PBS1X was checked by counting the cfu/ml at the beginning of the aggregation experiment and after a 16-h incubation. We performed experiments in three separate biological replicates, measured as three technical replicates each.

Hydrophobicity was determined by a salting out method as described by Misawa and Blaser (52). Sodium phosphate at 2 mm was used to make serial 2-fold dilutions of 4 m ammonium sulfate (25 μl each) to a final concentration of 0.00195 m in a U-bottomed 96-well plate. *C. difficile* strains were grown in BHIS agar, and a bacterial suspension in 2 mm sodium phosphate was used, and the *A*_600 nm_ was adjusted to 1.0. Twenty-five microliters of the bacterial suspensions were dispensed into each well, and the plate was incubated for 24 h statically in anaerobic conditions. The minimum concentration of ammonium sulfate allowing cells to aggregate defined the point of hydrophobicity. We performed experiments in three separate biological replicates, measured as three technical replicates each.

##### Biofilm on Abiotic Surface

This assay was performed as described (23, 53). Briefly *C. difficile* strains were grown in 5 ml overnight. Then 2 ml of BHIS were inoculated in low evaporation 24-well plates at a 1:100 dilution. The cultures were grown for 6 days, after which the supernatant was carefully removed, and the wells were washed with PBS. 1% crystal violet was added to the wells and incubated at room temperature for 20 min. The wells were then washed with PBS, and the remaining crystal violet was detached from the cells attached to the surface of the wells with methanol for 15 min at room temperature. The *A*_595 nm_ was measured in a spectrophotometer (BioTek, Swindon, UK). We performed experiments in three separate biological replicates, measured as three technical replicates each.

##### Adherence of C. difficile to Caco-2 IECs

Caco-2 cells grown in DMEM supplemented with 10% FCS and 1% penicillin-streptomycin (Gibco, Paisley, UK) were seeded at a density of 5 × 10^5^/well in a 24-well plate and grown for 9 days to obtain differentiated confluent monolayers (54). Cells were then co-cultured with 2.5 × 10^7^ cfu/ml FITC-labeled WT R20291, WT CD1426, or their respective flagellin glycosylation isogenic mutants for 1.5 h under anaerobic conditions. Bacterial adherence was assessed by flow cytometry and expressed as change in median fluorescent intensity of FITC. *C. difficile* flagellin-human TLR5 interaction HEK293 cells were kindly provided by Dr. D. Guilliano (University College London). Untransfected HEK293 cells were grown in DMEM supplemented with 10% FCS, 1% penicillin-streptomycin (Gibco), and 100 μg/ml Normocin (InvivoGen, Toulouse, France). TLR5-transfected HEK293 cells were grown in the same medium with the addition of 10 μg/ml Blasticidin (InvivoGen). Cells were seeded overnight at a density of 5 × 10^5^ cells/well in a 24-well plate and then co-cultured with WT and mutants at a multiplicity of infection of 10 for 8 h. Supernatants were collected, and IL-8 was measured by ELISA (Peprotech, London, UK) according to the manufacturer's instructions. We performed experiments in three separate biological replicates, measured as three technical replicates each.

##### Statistical Analysis

Data were analyzed by Tukey's multiple-comparison test using Prism software 9 version 4.0 (GraphPad Software, Inc., San Diego, CA). *p* < 0.05 was considered statistically significant.

## Author Contributions

E. V., M. B. E., A. D., and B. W. W. designed research; E. V., A. F.-P., L. F. D., and E. D. constructed mutants; E. V. and M. S. performed phenotypic assays; R. A. S. sequenced the mutants; P. H. and L. B. produced mass spectrometry data; E. V., S. L., P. H., and A. D. analyzed data; M. P., H. R. M., S. M. L., E. V., and B. W. W. wrote the manuscript; all authors edited the paper.

## Supplementary Material

Supplemental Data

## Footnotes

4 The abbreviations used are:

RT: ribotype
GT: glycosyltransferase
HexNAc: hexose *N*-acetylhexosamine
GlcNAc: *N*-acetylglucosamine
PTM: post-translational modification
TEM: transmission electron microscopy
IEC: intestinal epithelial cell
BisTris: 2-[bis(2-hydroxyethyl)amino]-2-(hydroxymethyl)propane-1,3-diol.
