# Supplementary material for: Role of Glycosyltransferases Modifying Type B Flagellin of Emerging Hypervirulent Clostridium difficile Lineages and Their Impact on Motility and Biofilm Formation
Source: J Biol Chem. 2016 Oct 4;291(49):25450–61. doi: 10.1074/jbc.M116.749523 (PMC5207246; doi:10.1074/jbc.M116.749523)
Supplement: Supplemental Data [file supp_291_49_25450__index.html]

Role of glycosyltransferases modifying type B flagellin of emerging hypervirulent Clostridium difficile lineages and their impact on motility and biofilm formation — Role of Glycosyltransferases Modifying Type B Flagellin of Emerging Hypervirulent Clostridium difficile Lineages and Their Impact on Motility and Biofilm Formation — C. difficile Type B Flagellin Affects Motility and Biofilm — Supplemental Data 

# Role of Glycosyltransferases Modifying Type B Flagellin of Emerging Hypervirulent *Clostridium difficile* Lineages and Their Impact on Motility and Biofilm Formation

## Supplemental Data

- Supplemental material (.pdf, 424 KB) - Supplemental material
