# Supplementary material for: Role of Glycosyltransferases Modifying Type B Flagellin of Emerging Hypervirulent Clostridium difficile Lineages and Their Impact on Motility and Biofilm Formation
Source: J Biol Chem. 2016 Oct 4;291(49):25450–61. doi: 10.1074/jbc.M116.749523 (PMC5207246; doi:10.1074/jbc.M116.749523)
Supplement: Supplemental Data [file 10.1074_M116.749523_jbc.M116.749523-1.pdf]

Role of glycosyltransferases modifying type B flagellin of emerging hypervirulent *Clostridium difficile* lineages and their impact on motility and adhesion

Esmeralda Valiente[1], Laura Bouché[2], Paul Hitchen[2], Alexandra Faulds-Pain[1], Mario Songane[3], Lisa F. Dawson[1], Elizabeth Donahue[1], Richard A. Stabler[1], Maria Panico[2], Howard R. Morris[2,5], Mona Bajaj-Elliott[3], Susan M. Logan[4], Anne Dell[2] and Brendan W. Wren[1*]

Supplementary figure 1

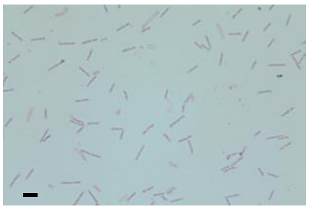

R20291

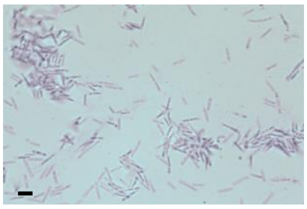

GT1

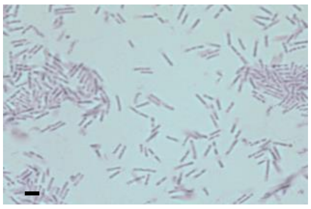

GT2

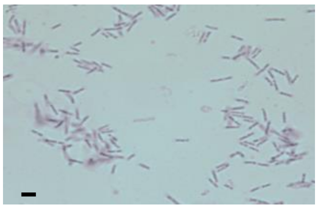

GT3

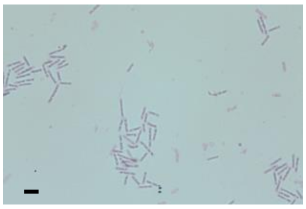

CDR20291_0245

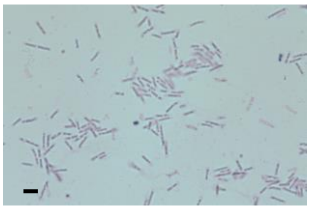

CDR20291_0246

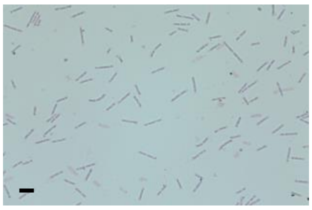

R20291*fliC*

Supplementary figure 2

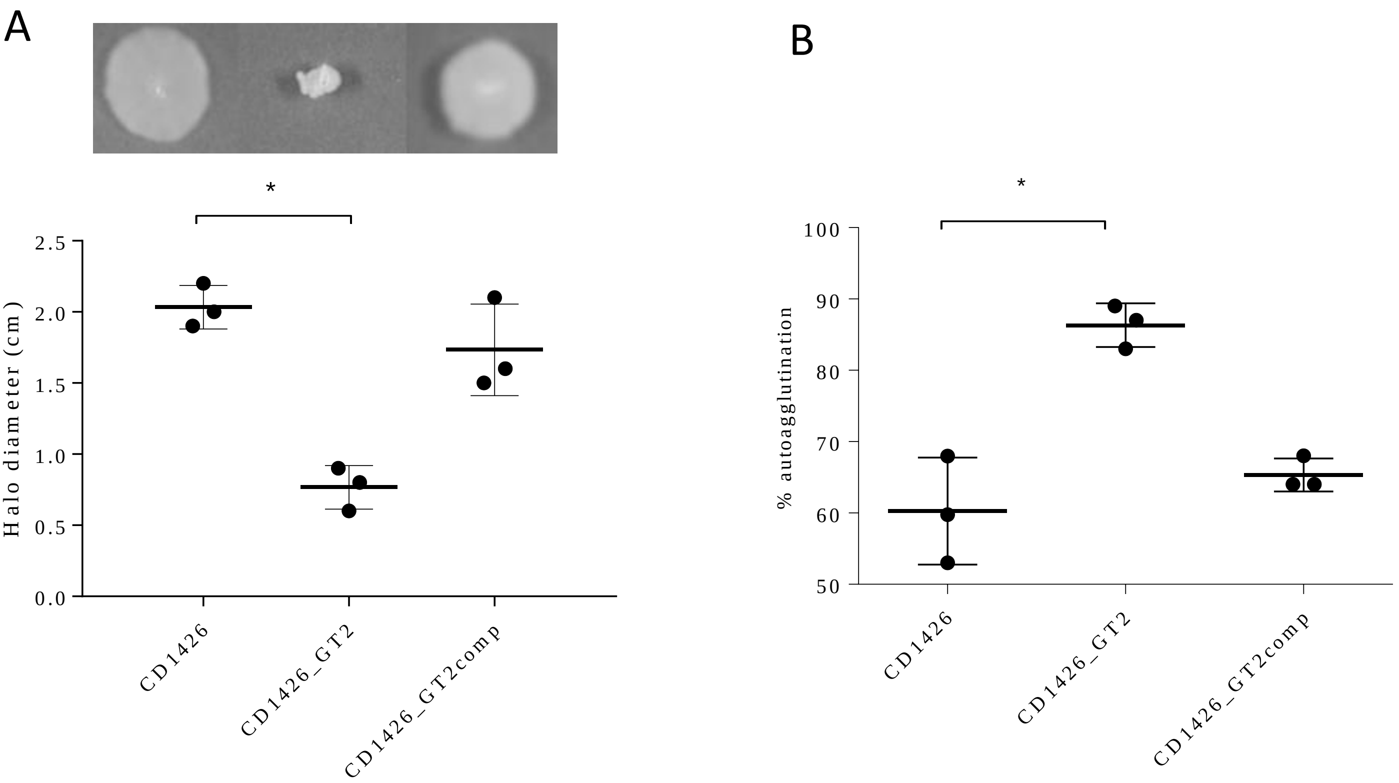

Supplementary table 1. List of oligonucleotides used in this study

Primers used for screening mutants and make complements	Nucleotide sequence (5'—3')
*fliC*F	GGGAAGAAACGTAAATGCACA
*fliC*R	TTGTGCCCCTAATTTTGCTC
GT1F	TCCTATGCTAAAGGTGAATGGT T
GT1R	TCCCTAAATCTACTTGTGCTTG T
GT2F	ACACCAATAGTGGTTAATGA
GT2R	TATGTTTTCACCAGTAGCCA
GT3F	ACCTGTGTAAATGATGAAAG
GT3R	GCATCTTGATGTAAGTATACCT
CDR20291_0245F	TGCGAAAAAGATTTGTGATGA
CDR20291_0245R	CCATCTTGTGAGGCTTCCTT
CDR20291_0246F	CAGTAGAAATAACTACATC
CDR20291_0246R	CAGTAGAAATAACTACATC
GT2-EBS1δ	CAGATTGTACAAATGTGGTGAT AACAGATAAGTCGATCCAACT AACTTACCTTTCTTTGT
GT2-EBS2	TGAACGCAAGTTTCTAATTTCG GTTGAAGATCGATAGAGGAAA GTGTCT
GT2-IBS	AAAAGCTTATAATTATCCTTAT CTTCCGATGGAGGTGCGCCCAG ATAGGGTG
GT3-EBS1δ	CAGATTGTACAAATGTGGTGAT AACAGATAAGTCGAGGAATGT AACTTACCTTTCTTTGT
GT3-EBS2	TGAACGCAAGTTTCTAATTTCG GTTTAAAGTCGATAGAGGAAA GTGTCT
GT3-IBS	AAAAAGCTTATAATTATCCTTA CTTTACGAGGAAGTGCGCCCAG ATAGGGTG
EBS universal	CGAAATTAGAAACTTGCGTTCA GTAAAC
RAM-F	ACGCGTTATATTGATAAAAATA ATAATAGTGGG
RAM-R	ACGCGTGCGACTCATAGAATTA TTTCCTCCCG
GT1EcoRI_F	ATAGAATTCATTGGATATAGAA GAAAGGG
GT1XbaI_R	ATATCTAGATCTATTTGATTAT ATGTC
GT2NdeI_F	TATGAATACACCAATAGTGGT *
GT2 EcoRI_R	ATCGAATTCACAAGACATTCCT CATAAAGT
GT3_EcoRI_F	ATAGAATTCAATGAATCACCAA TAGTG
GT3_XbaI_R	ATATCTAGAGTCCTCAGTGAAT

	TTTCC
CDR20291_0245EcoRI_F	ATAGAATTCATGAAAAGATTA ATGATTTTAGG
CDR20291_0245XbaI_R	ATATCTAGATTAAAAATTTTCT ATCAT
CDR20291_0246EcoRI_F	ATAGAATTCTATGATAGATATG AAGCTA
CDR20291_0246XbaI_R	ATATCTAGATTGCTTTATTTGA TTCTA
Primers used for RT-PCR	
1F	GTCTCAAAGACCACTATATTCT G
1R	GGTAATATATCATTGGTTTATG G
2F	CCAAGCCACTGATAAAAACTG
2R	CAAGACCCTGCTCTAT
3F	GGTTTATATGGAATGACAAGC
3R	ACCTTTCTTCTTCAAATAGCT
4F	GACAGTATTAGAGATGGATAG AG
4R	CAATAGAATCGGCTCCT
5F	GTGAATTAGAAGAATTTGATAC TG
5R	GCAATAGAATCGGCTCCTCT
6F	GATGAAATCGAAGACTATCAG C
6R	CATAAGTTTCACATGCAGG
7F	GGCAAATACTTAGACGAGG
7R	GAGTATCAATTCTATTATCAC
8F	GATGAGAGACAGGATTGTGG
8R	CTCCCTGGCAATCTTGTAACC
9F	GATAGAATTAAAAGAACC
9R	GCCTTACTTCATTTCCAC
luxS-F	GTACTTGATGGAGTAAAGG
luxS-R	TTGCATATTCCTTTGCC
Int-F	GTGTCTTGTATTGGGGG
Int-R	CCAGTATCAAGCCAAGCC
*fliC*F	GGGAAGAAACGTAAATGCACA
*fliC*R	TTGTGCCCCTAATTTTGCTC

*5’end is phosphorilated

SUPPLEMENTARY FIGURE 1. Optical microscopy images of R20291 and its corresponding flagellin modification mutants showing cell aggregation in the mutants. The bar corresponds to 1 µm.

SUPPLEMENTARY FIGURE 2. Motility and cell aggregation assays in RT 023 and GT2 mutant. A. Motility in CD1426 (RT023) and corresponding modification mutant and complement; B. Cell aggregation in CD1426 and corresponding modification mutant and complement. Data presented as the mean ± SEM from 3 independent experiments with three technical replicates each. Significant differences are marked with asterisk; $p < 0.05$.
